# A thirty year, fine-scale, characterization of area burned in Canadian forests shows evidence of regionally increasing trends in the last decade

**DOI:** 10.1371/journal.pone.0197218

**Published:** 2018-05-22

**Authors:** Nicholas C. Coops, Txomin Hermosilla, Michael A. Wulder, Joanne C. White, Douglas K. Bolton

**Affiliations:** 1 Integrated Remote Sensing Studio, Department of Forest Resources Management, University of British Columbia, Vancouver, British Columbia, Canada; 2 Canadian Forest Service (Pacific Forestry Centre), Natural Resources Canada, Victoria, British Columbia, Canada; University of Saskatchewan, CANADA

## Abstract

Fire as a dominant disturbance has profound implications on the terrestrial carbon cycle. We present the first ever multi-decadal, spatially-explicit, 30 meter assessment of fire regimes across the forested ecoregions of Canada at an annual time-step. From 1985 to 2015, 51 Mha burned, impacting over 6.5% of forested ecosystems. Mean annual area burned was 1,651,818 ha and varied markedly (σ = 1,116,119), with 25% of the total area burned occurring in three years: 1989, 1995, and 2015. Boreal forest types contained 98% of the total area burned, with the conifer-dominated Boreal Shield containing one-third of all burned area. While results confirm no significant national trend in burned area for the period of 1985 to 2015, a significant national increasing trend (α = 0.05) of 11% per year was evident for the past decade (2006 to 2015). Regionally, a significant increasing trend in total burned area from 1985 to 2015 was observed in the Montane Cordillera (2.4% increase per year), while the Taiga Plains and Taiga Shield West displayed significant increasing trends from 2006 to 2015 (26.1% and 12.7% increases per year, respectively). The Atlantic Maritime, which had the lowest burned area of all ecozones (0.01% burned per year), was the only ecozone to display a significant negative trend (2.4% decrease per year) from 1985 to 2015. Given the century-long fire return intervals in many of these ecozones, and large annual variability in burned area, short-term trends need to be interpreted with caution. Additional interpretive cautions are related to year used for trend initiation and the nature and extents of spatial regionalizations used for summarizing findings. The results of our analysis provide a baseline for monitoring future national and regional trends in burned area and offer spatially and temporally detailed insights to inform science, policy, and management.

## Introduction

Wildfires are a critical process that occur across most forested ecosystems and play a pivotal role in the terrestrial and atmospheric carbon cycle [1]. Globally, approximately 3.5 million hectares of forest burn each year [1], corresponding to about 2.5% of the dry land mass of the planet, with most regions having a fire season that lasts less than six months [2]. The CO_2_ emissions associated with fires have been shown to exceed 50% of the emissions estimated from fossil fuel combustion [3]; however this phenomena is highly variable temporally. Wildfire is also a critical disturbance process in forest ecology, driving spatial legacies that can persist from decades to millennia, with implications to forest structure, age class distribution, composition as well as impacts to carbon, water and nutrient cycles [4]. Across Canada, which represents ~10% of the world’s forest, fire is the dominant stand-replacing disturbance impacting forested ecosystems [4]; however, fires are highly variable in terms of size and frequency [5,6], due to broad-scale climatic, biological, and physiographical conditions [6–8] as well as anthropogenic activities. Previous studies showed that around 8000 fires burned approximately 1.8 million hectares of forest per year from 1959 to 1997, with most of the area burned in boreal forest ecosystems [9] and a mean fire return interval of 180 years [10].

Under a changing climate, patterns in a number of wildfire characteristics such as area burned, size, occurrence, and seasonality are expected to change as the weather becomes more fire conducive [11], although the increase in area burned is expected to be gradual [12]. Already, recent shifts in area burned and seasonality are showing patterns consistent with these warmer climate patterns [13]. A range of fire event descriptors such as fire location, extent, timing and magnitude (which can be a measure of disturbance intensity or severity), provide overall context for characterizing a fire regime [14],[15]. In contrast to individual wildfire events, a regime describes the temporal and spatial characterization of these disturbance events through a specific landscape and defined period of time [16]. By definition, therefore, a fire regime cannot be inferred from a single fire, but rather many events are required to explain the spatial and temporal patterns and offer insight into fire behavior, spatial distributions of fire size, severity, annual area burned and temporal dynamics such as seasonality. Understanding how fire regimes vary across Canada is of significant importance from a variety of perspectives: scientists understanding broad-scale effects of fire on landscape structure and impacts associated with climate change; managers charged with undertaking sustainable forest management practices and assessing broad-scale biodiversity patterns, especially in areas where fire is the most prevalent natural disturbance [9]; and government agencies responsible for monitoring and accounting for carbon fluxes in Canadian forests [17].

Information on fire event characteristics over Canada’s forested ecosystems has conventionally been derived in recent times from a combination of data sources, primarily, GPS (i.e. fire boundaries recorded from aircraft or helicopters), air photo interpretation, and satellite imagery. Given that the mandate for forest management in Canada is the purview of provincial and territorial agencies, data are compiled by each responsible jurisdiction and shared through the Canadian Wildland Fire Information System. As Canadian forests are composed of both managed and unmanaged land crossing provincial and territorial borders, multiple jurisdictions are involved in stewardship; leading to issues in both temporal and spatial compilations [5,9]. The implication of these spatial and temporal inconsistencies in national-level fire information requires users to often only consider larger, and more, severe fires, potentially leading to bias in national level trends and statistics. As a result, a number of agencies are turning to higher spatial resolution remote sensing imagery (< 1km) to better discriminate fire boundaries and better understand the burned and unburned mosaic remaining on the forested landscape. The FireMARS system in Canada for example is moving towards delineation of burned area polygons mapped nationally on an annual basis through the integration of data from fine and coarse spatial resolution satellite data from 2004 onwards (http://www.nrcan.gc.ca/forests/fire-insects-disturbances/fire/13159) [18].

The opening of the Landsat archive and the associated processing and analysis opportunities [19] enables the characterization of the entire area of interest with the same information source, thereby allowing for the transparent, systematic, and repeatable production of spatially exhaustive information products [20–22]. As a result, the type, area, severity and timing of disturbance events can be quantified [21,23–27], often at higher spatial resolution than previous approaches, providing a consistent dataset for land cover change and disturbance characterization [28].

As a result, fire events can now be comprehensively detected and characterised across the entire forested land-base of Canada, availing upon the 30 m spatial resolution data in the Landsat archive. This unprecedented capacity to systematically detect burned areas allows for detailed temporal and spatial analysis of wildfire: providing a marked improvement in our understanding of where fires have occurred, both spatially and temporally, the characteristics of these wildfires, and changes in these characteristics over time across the forested ecosystems in Canada. In this paper we demonstrate how Landsat time-series information can be used to systematically analyze, across the entire forested landbase of Canada, the year and spatial extent of fires from 1985–2015. To do so, we compare burned area estimates across Canadian ecozones and predefined homogenous fire regimes, to examine if any temporal trends in size or frequency are evident over the time period, and demonstrate how these refined estimates of the burned area inform upon national fire regimes for Canada.

## Materials and methods

### Study area

Canada’s forests represent approximately 10% of global forests and 30% of global boreal forests. In 2014, Canada’s forest industry contributed 20.1 billion dollars to Canada’s GDP and accounted for 30.7 billion dollars’ worth of exports [29]. Natural disturbances such as fire, insects, disease, and drought play an important role in the dynamics of Canada’s forests. While these disturbances vary in their spatial extent, temporal frequency, and severity, wildfire has a unique impact on the landscape as it has both positive and negative effects [30]. Our analysis focuses on the forest supporting ecosystems of Canada, as defined by those ecozones with a majority of forest vegetation and/or forestry land use (as opposed to areas dominated by agricultural or other land uses, such as the Prairie, Mixedwood Plains or the southern Arctic ecozones). Ecozones are higher order ecosystems that represent the most generalized level of an expert-based, hierarchical, ecological framework for Canada [31]. Ecozones are defined as areas expressing similar geology, topography, soil, vegetation, climate, wildlife, hydrology, and land use. The twelve forested ecozones of Canada occupy approximately 650 million ha [32], including over 347 million ha of treed and other wooded land (http://nfi.nfis.org), along with wetlands and lakes, among other cover types. As a contrasting scale of analysis we also examine the pattern of fire events using a more recently developed regionalization proposed by Boulanger et al. [9] of homogeneous fire regime units across Canada. In total 16 fire regime areas were defined using a spatial clustering of 40 × 40 km forested grid cells over the country based on fire occurrence data of fires > 1ha from 1959–2008 from Canada’s National Fire Database (CNFDB), which is a multi-source compilation of fire polygon data collected by fire management agencies. The fire regimes offered a fire-focused spatial regionalization from a snapshot of recent fire conditions whereas the ecological units capture a broader range of environmental conditions including geology and terrain, and as a result, are expected to capture longer term and historical ecological conditions.

### Fire data

Canada is covered by over 1200 Landsat scenes (path / rows) of the Landsat Worldwide Referencing System (WRS-2) [33]. Candidate images for compositing included all images with less than 70% cloud cover from the United States Geological Survey (USGS) archive of Level-1-Terrain-Corrected (L1T) Landsat Thematic Mapper (TM), Landsat Enhanced Thematic Mapper Plus (ETM+) and Landsat Operational Land Imager (OLI) data acquired August 1^st^ ± 30 days (corresponding to the average growing season) from 1984 to 2015. Approximately 100,000 images were ultimately used to produce annual best-available-pixel (BAP) composites. As summarized in White et al. [22], all eligible data were converted to surface reflectance values using Landsat Ecosystem Disturbance Adaptive Processing System (LEDAPS) algorithm [34]. Clouds, and their shadows are detected and masked using the Fmask algorithm [35].

Annual BAP image composites were then created using the pixel-scoring functions described in White et al. [22]. Following the Composite2Change (C2C) approach of Hermosilla et al. [36,37], disturbance events were detected using times series spectral trend analysis protocol, which involved noise detection, breakpoint identification, contextual analysis, and generation of gap-free composites [38]. Change events in the trajectory of a pixel series through time were detected using a bottom-up breakpoint detection algorithm using Normalized Burn Ratio (NBR) values [39]. An object-based image analysis approach was applied where change objects are created by the spatial aggregation of changed pixels with the same year of occurrence and duration of event. A set of metrics are derived from the Landsat spectral trend analysis to characterize the change events, as well as pre- and post-change conditions, which are used to attribute change events. Additionally, to enable attribution of change events to a change type, a set of spectral metrics are computed for selected Landsat bands and for several spectral indices (NBR, and Tasseled Cap Brightness (TCB), Greenness (TCG), and Wetness (TCW) [40]. The geometric complexity of the change objects are described using the area, perimeter, compactness and fractal dimension [41]. This spatial and spectral information is utilized to label the changes found through the Landsat spectral trend analysis, as described in Hermosilla et al. [42]. We define four change classes of relevance: fire, harvest, road, and land cover condition change.

Change attribution accuracy was assessed using an independent evaluation sample set, where reference classes were manually interpreted using the Landsat annual image composites and high spatial resolution imagery from Google Earth. Ancillary data, including the CNFDB, were used to support the visual interpretation of change attribution. Representative reference samples were allocated using a restricted random selection [37], which insured the spatial homogeneity of the samples, followed by redistribution and inclusion of additional samples to maintain an appropriate number, with respect to the variability in each class. Change objects are attributed to one of the four change attribution classes using a random forest classifier [43] with an overall accuracy of 92%. Fire shows the lowest commission (2%) error and amongst the lowest omission (7%) error and strong separability with harvesting and infrastructure classes [37]. Using this approach every detected fire is regarded as a discrete fire patch both in space and time. The annual nature of the BAP composites utilized restricts this analysis to year as the timing of fire events (within the fire season) is not captured.

### Analysis approach

**Fig 1:**
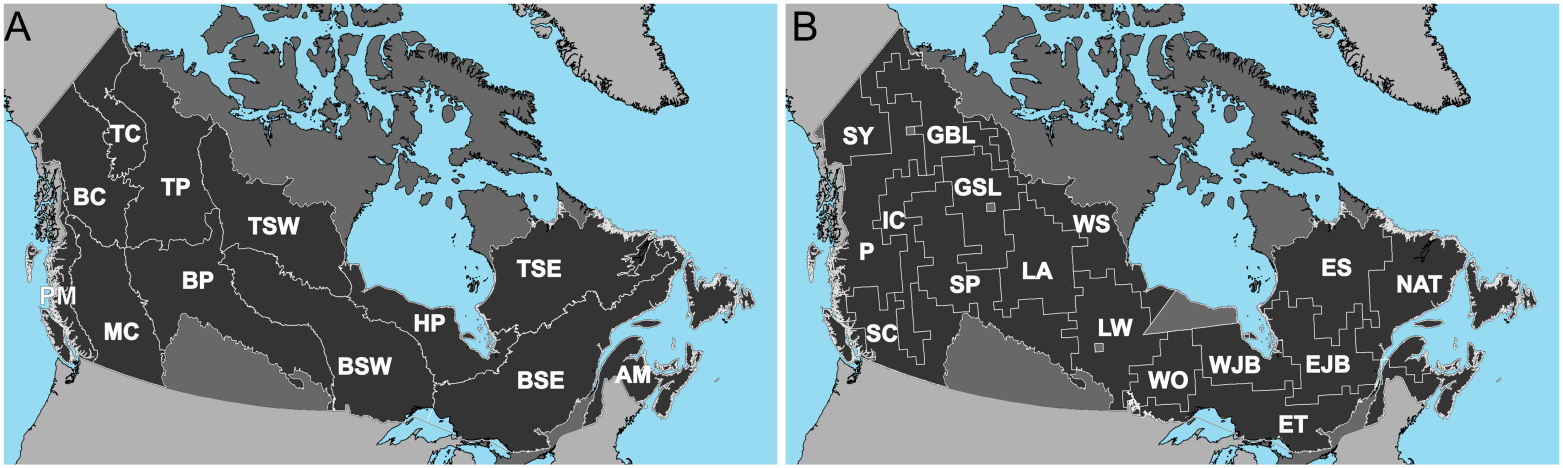
Ecozones and Boulanger et al (2012) regionalization. Ecozone names: Atlantic Maritime (AM), Pacific Maritime (AM), Boreal Shield East (BSE), Boreal Shield West (BSW), Taiga Shield East (TSE), Taiga Shield West (TSW), Boreal Cordillera (BC), Montane Cordillera (MC), Taiga Cordillera (TC), Boreal Plains (BP), Taiga Plains (TP), and Hudson Plains (HP). Fire regime names: Eastern Subarctic zone (ES), Great Slave Lake zone (GSL), Southern Cordillera zone (SC), Lake Winnipeg zone (LW), Eastern Temperate zone (ET), Lake Athabasca zone (LA), Eastern James Bay zone (EJB), Interior Cordillera zone (IC), Western Ontario zone (WO), Western Subarctic zone (WS), Great Bear Lake zone (GBL), Western James Bay zone (WJB), Southwestern Yukon zone (SY), North Atlantic zone (NAT), Southern Prairies zone (SP), and Pacific zone (P). Map generated in ArcGIS 10.1. (http://www.esri.com/software/arcgis/arcgis-for-desktop). Ecozone boundaries available via creative commons.

To examine and report on burned patches and regimes we utilised the two stratifications of Canada (Fig 1) and a 50 × 50 km grid for spatial generalization and comparative purposes. At the ecozone scale we divide the Boreal and Taiga Shield ecozones into west and east units, as established by Kurz et al. [44] and supported by Frazier et al. [45], who demonstrated the colder and drier climate in the western half of the Boreal Shield ecozone resulted in different disturbance and ecological processes compared to the eastern portion.

**Fig 2:**
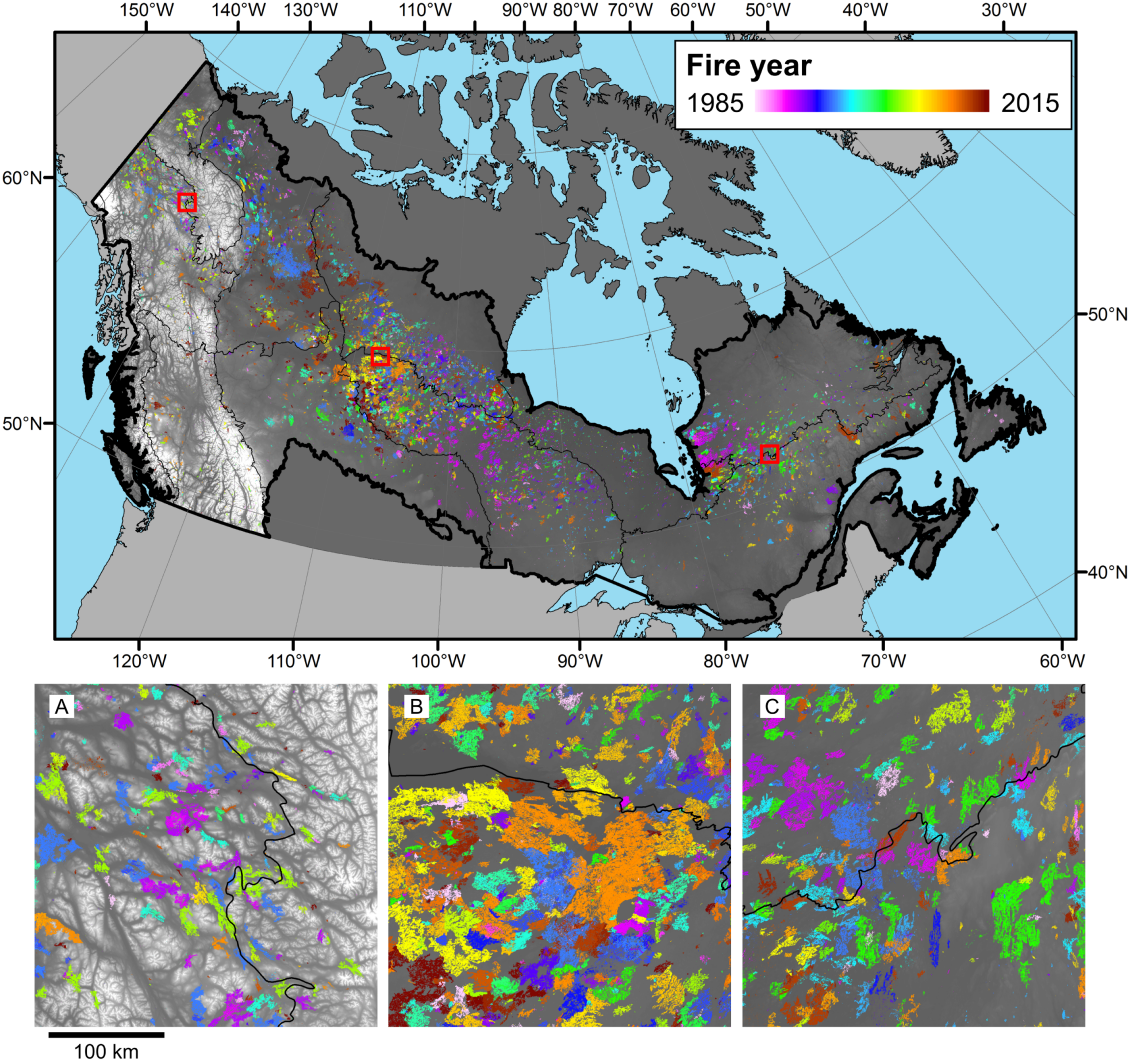
Annual distribution of burned areas (1985–2015) in Canada’s forested ecosystems. Insets show details of the fire events in three sub-areas (ordered west to east). Ecozone boundaries are also shown (as per Fig 1). Map generated in ArcGIS 10.1 (http://www.esri.com/software/arcgis/arcgis-for-desktop). Ecozone boundaries available via creative commons.

**Fig 3:**
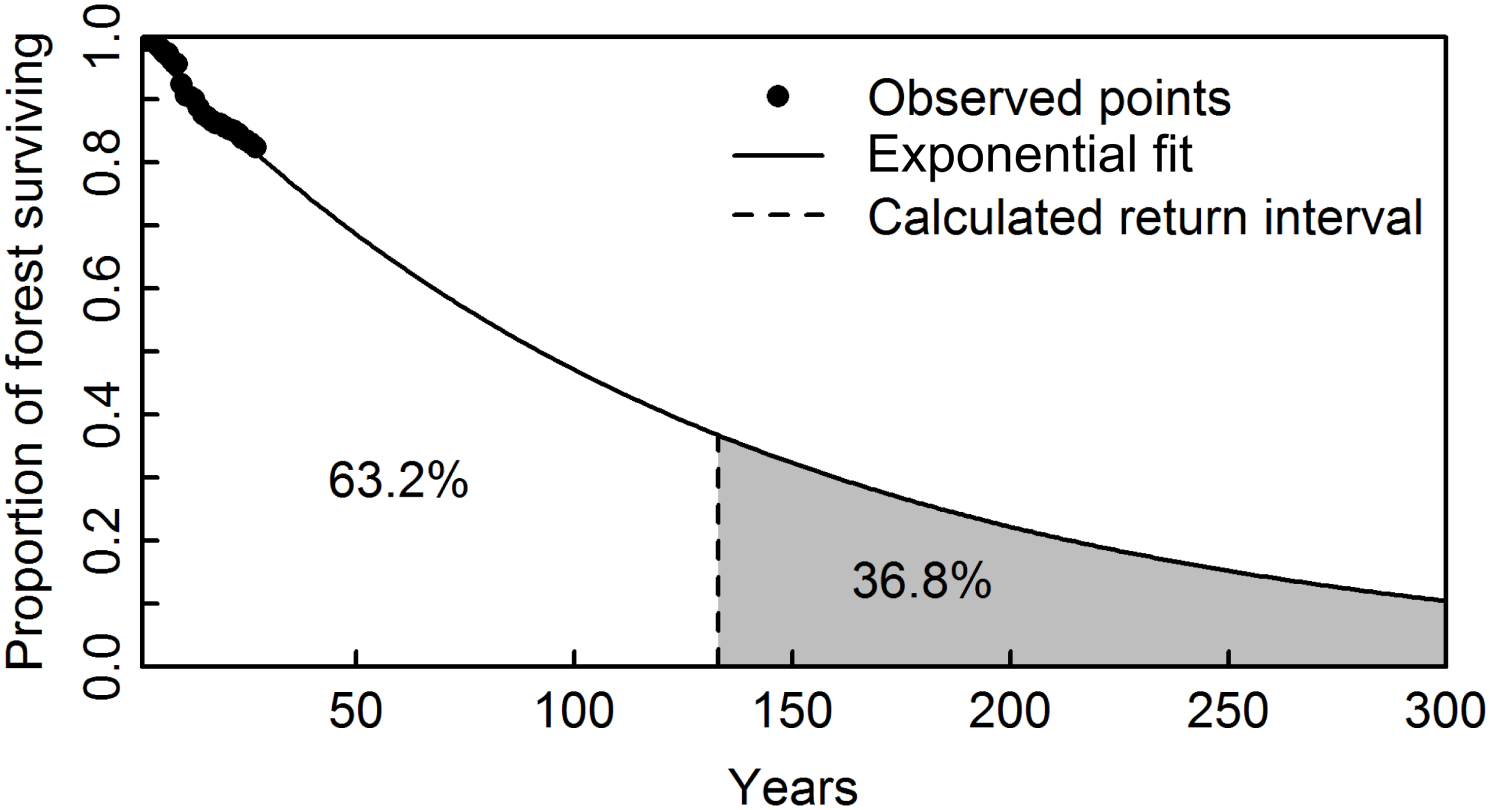
Exponential fitting for fire return interval computation in the Taiga Shield West ecozone.

Fire regimes were assessed for each ecozone or fire regime area using a set of key criteria [9,14]. Burned area size was computed directly from the Landsat derived fire objects (patches) on an annual basis (Fig 2). The rate at which the forested landscape was burned was calculated as the proportion of annual area burned by the total area available to burn (all remaining pixels in the forested ecozone / fire regime reduced by snow/ice, agricultural areas, and water). Fire return intervals, a key indicator for fire regime characterisation, were estimated for each of the forested ecozones / fire regimes. The mean fire return interval is the mean of a negative exponential fit to the proportion of surviving forest (by area; following Van Wagner [46]) and corresponds to the average time between two fires at the same location, or 1/burn rate. The fire return interval informs on the time between fires within a defined spatial area (e.g. ecozone) and enables regional comparisons. Fig 3 shows an example of the fire return interval calculation for Taiga Shield West ecozone.

All statistical analysis was completed in R [47]. First, we analysed the total area burned across the forested ecozones and fire regimes, by year, and then by five year epochs within 50 × 50 km grid cells. Non-parametric tests were applied to examine if temporal trends existed in the area burned by ecozone and fire regime. Slopes of the area burned through time were estimated using the Theil-Sen non-parametric trend slope estimator and significance tests were performed using the Mann-Kendall trend test in the wq package for R [48]. Similarly, we tested if the number of large fire patches (> 200 ha) changed through time for each ecozone and fire regime.

## Results

Over the entire analysis period, from 1985 to 2015, a total of 51,206,361 ha in Canada’s forested ecozones burned, representing 6.5% of Canada’s forested ecosystems. The total area burned annually varied markedly. On average, the annual total area burned was 1,651,818 ha (with a standard deviation of 1,116,119 ha); however in some years, the total area burned greatly exceeded this average. For example, fires in 1989, 1995, and 2015 accounted for 25% of the total area burned from 1985 to 2015 (6.8%, 11.6%, and 6.1% respectively for 1989, 1995, and 2015). The total area burned in 1989, 1995, and 2015 was greater than the area burned in 14 other years combined (1985–1988, 1991–1993, 1997, 2000, 2001, 2003, and 2007–2009).

**Fig 4:**
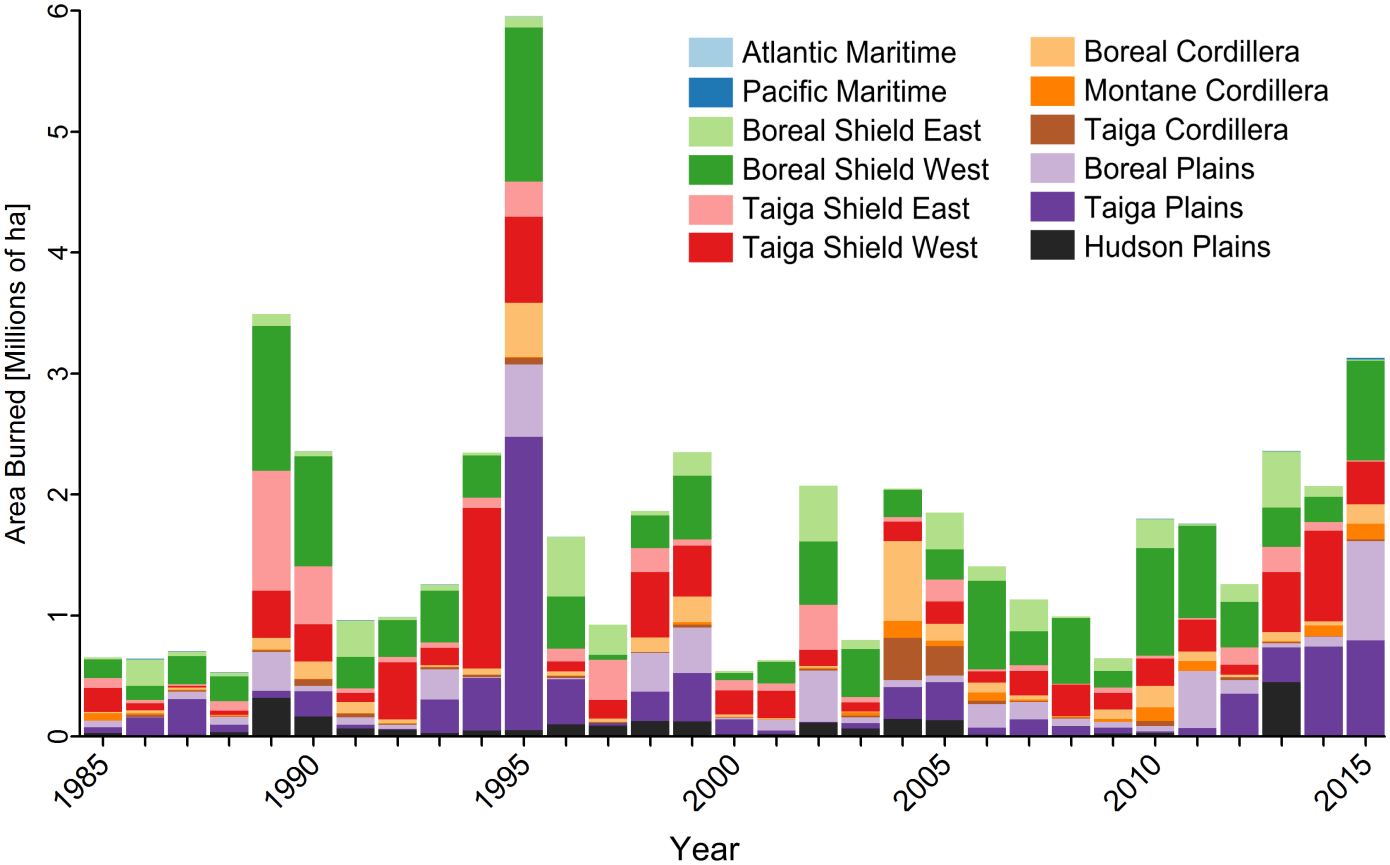
Annual distribution of total area burned (1985–2015), by ecozone.

The total area burned also varied markedly by ecozone and fire regime over the 30-year analysis period (Table 1 for ecozone, not shown for fire regime). Percent annual area burned (PAAB) was highest in the Taiga Shield West (0.68% yr^-1^) followed by the Boreal Shield West (0.67% yr^-1^), Taiga Plains (0.52% yr^-1^), Boreal Plains (0.30% yr^-1^), Taiga Shield East (0.24% yr^-1^), and Boreal Cordillera (0.23% yr^-1^). Compared to the boreal ecozones, the coastal ecozones in the west (Pacific Maritime) and east (Atlantic Maritime) had markedly lower PAAB (0.01% yr^-1^). In 1989, the area burned was predominantly in the Boreal Shield West and Taiga Shield East, whereas in 1995, the largest areas burned were concentrated in the Boreal Shield West and Taiga Plains (Fig 4). Although nearly half of area burned in 1995 was in the Taiga Plains ecozone, this ecozone represented only 12.9% of burned patches for 1995, indicating that the burned area in this ecozone occurred in relatively large patches. By contrast, the Taiga Shield West accounted for 11.9% of the total area burned in 1995 and 47.6% of the total burned patches, suggesting a greater frequency of small fire patches in this ecozone. Similarly, although the total area burned in 1990 represented only 40% of the total area burned in 1995, both years had a similar number of burned patches (1990 = 48,699 patches; 1995 = 53,499 patches).

**Table 1:** Average burned area, Theil Sen Test results, and estimated fire return intervals. Slopes only displayed for significant trends (p < 0.05). Slope percentages are relative to average annual area burned.

Zone	Average [ha yr^-1^]	Standard deviation [ha yr^-1^]	Percent Annual Area Burned [% yr^-1^]	Slope [ha yr^-1^] (calculated using Theil Sen test)			Fire return interval [yrs]
				2006–2015	1996–2015	1985–2015	
Atlantic Maritime	875.5	1,671.3	0.01			-20.6 (-2.4%)	> 5,000
Boreal Cordillera	94,360.0	138,114.7	0.23				439
Boreal Plains	158,449.4	198,607.5	0.30				369
Boreal Shield East	136,875.5	145,672.9	0.15				685
Boreal Shield West	432,440.6	319,983.3	0.67				141
Hudson Plains	75,545.1	96,601.0	0.22				429
Montane Cordillera	29,332.3	40,335.6	0.07		3207.23 (8.1%)	709.3 (2.4%)	> 2,000
Pacific Maritime	1,401.7	2,590.1	0.01		46.49 (3.2%)		> 5,000
Taiga Cordillera	33,619.7	73,066.8	0.14				709
Taiga Plains	269,631.2	446,484.9	0.52	66,548.5 (26.1%)			188
Taiga Shield East	136,524.7	197,415.5	0.24		-4990.73 (-4.8%)		340
Taiga Shield West	282,762.3	270,592.2	0.68	36,129.83 (12.7%)			139
Total	1,651,818.1	1,116,119.4	0.31	186,158 (11.2%)			321

**Fig 5:**
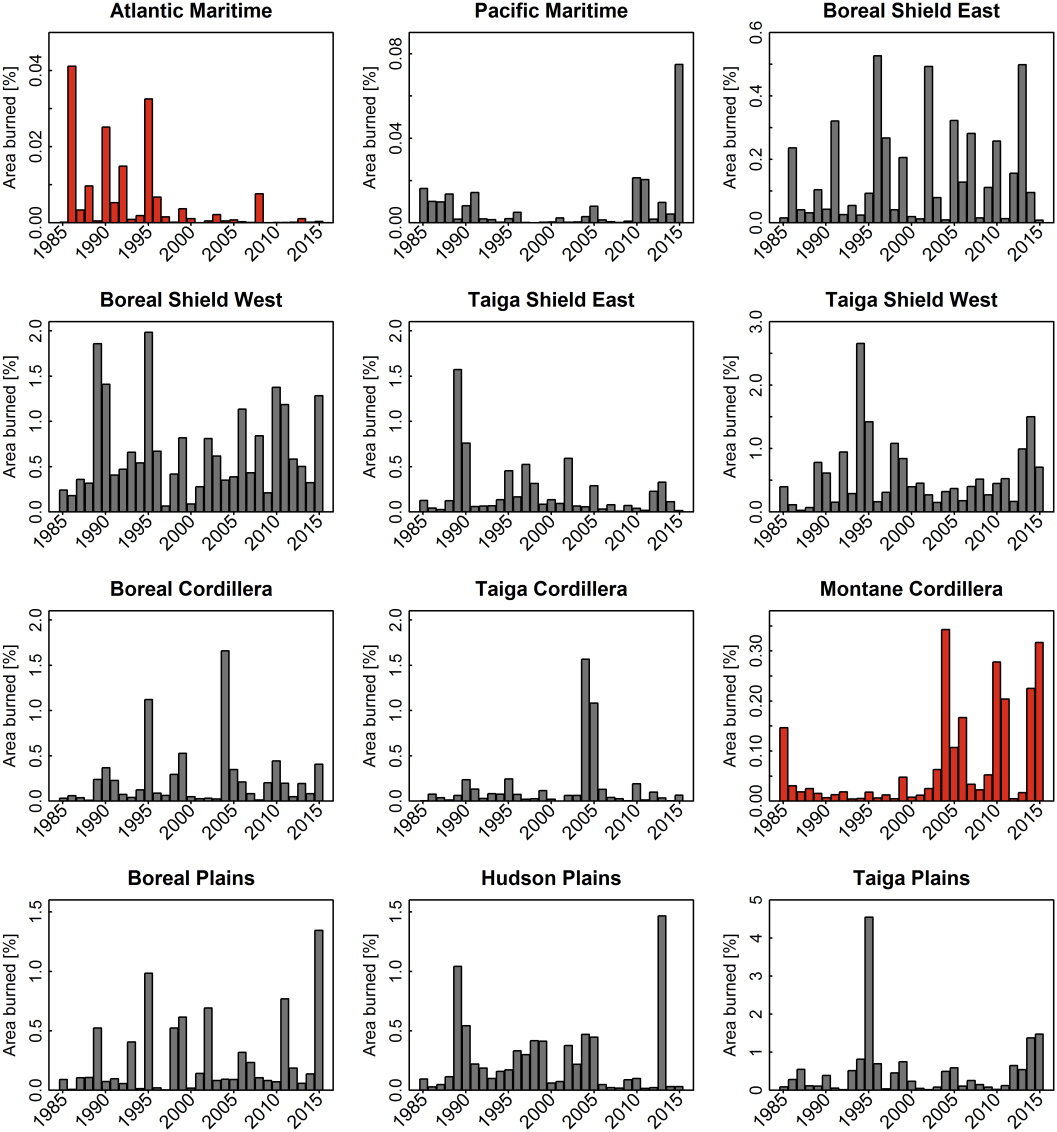
Histograms of percent annual area burned for each ecozone from 1985 to 2015. Montane Cordillera and Atlantic Maritime (shaded red) were the only ecozones to display a significant trend in percent area burned from 1985 to 2015 using the Thiel Sen test (α = 0.05).

**Fig 6:**
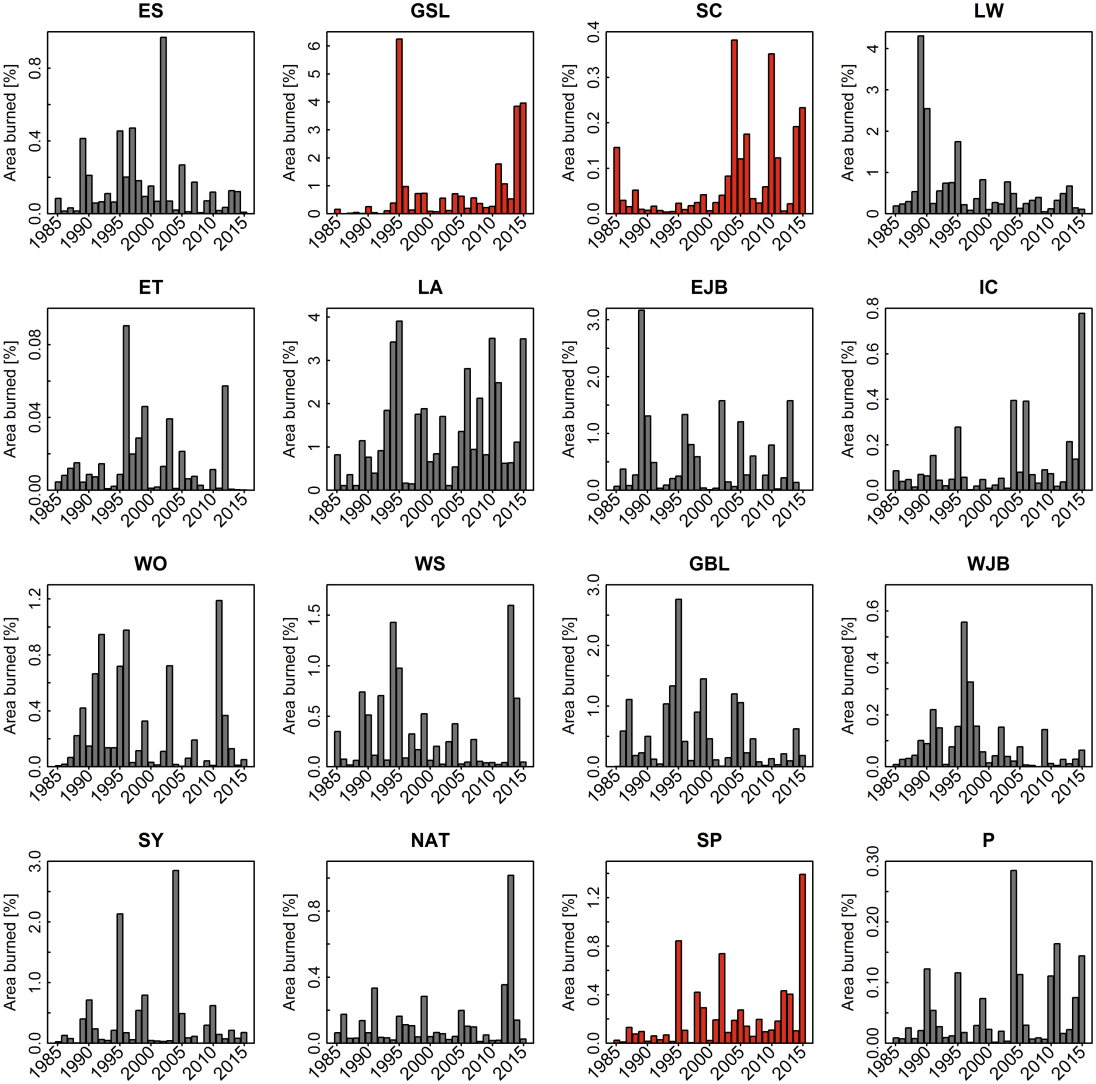
Histograms of percent area burned for each fire regime area from 1985 to 2015. GSL, SC and SP (shaded red) were the only fire regime areas to display a significant trend in percent area burned from 1985 to 2015 using the Thiel Sen test (α = 0.05).

The percent area burned annually by ecozone and fire regime area is presented in Figs 5 and 6, which show the general lack of trend over the analysis period in terms of area burned. We examined results of Theil Sen test for temporal trends in burned area, by ecozone and fire regime over the 30-year analysis period and found limited significant trends (α = 0.05, Table 1, Fig 5). Over the entire 30-year period, only the Atlantic Maritime and Montane Cordillera ecozones exhibited statistically significant trends in annual area burned, with a slight decrease (2.4% yr^-1^) and a slight increase (2.4% yr^-1^), respectively. As the annual burn rate was lowest in the Atlantic Maritime (0.01% yr^-1^), a decrease of 2.4% yr^-1^ only represents a decrease of 20.6 ha yr^-1^, while the increase of 2.4% yr^-1^ in the Montane Cordillera represents an increase of 709.3 ha yr^-1^. For the 2006–2015 period, there were significant increasing trends in burned area in both the Taiga Plains (26.1% yr^-1^) and the Taiga Shield West (12.7% yr^-1^), as well as an increase of 11% yr^-1^ nationally (increase of 180,000 ha yr^-1^ nationally). By homogeneous fire regime regionalization 3 of the 16 units (GSL, SC and SP) showed significant trends in burned area from 1985 to 2015, all increasing by 2.8–2.9% yr^-1^, and all occurring within western Canada, essentially overlapping Taiga Shield West and Montane Cordillera ecozones. Only the Montane Cordillera ecozone had a significant increase in the number of large (> 200 ha) burned patches from 1985 to 2015 (3.5% yr^-1^). Alternatively, four of the 16 fire regimes had a significant increase in large burned patches from 1985 to 2015 (GSL, LA, SC, and SP had increases of 3.6, 2.7, 4.5, and 4.7% yr^-1^, respectively), while two fire regimes had a significant decrease in the number of large patches (3.1% yr^-1^ for ET and 4% yr^-1^ for WJB).

**Fig 7:**
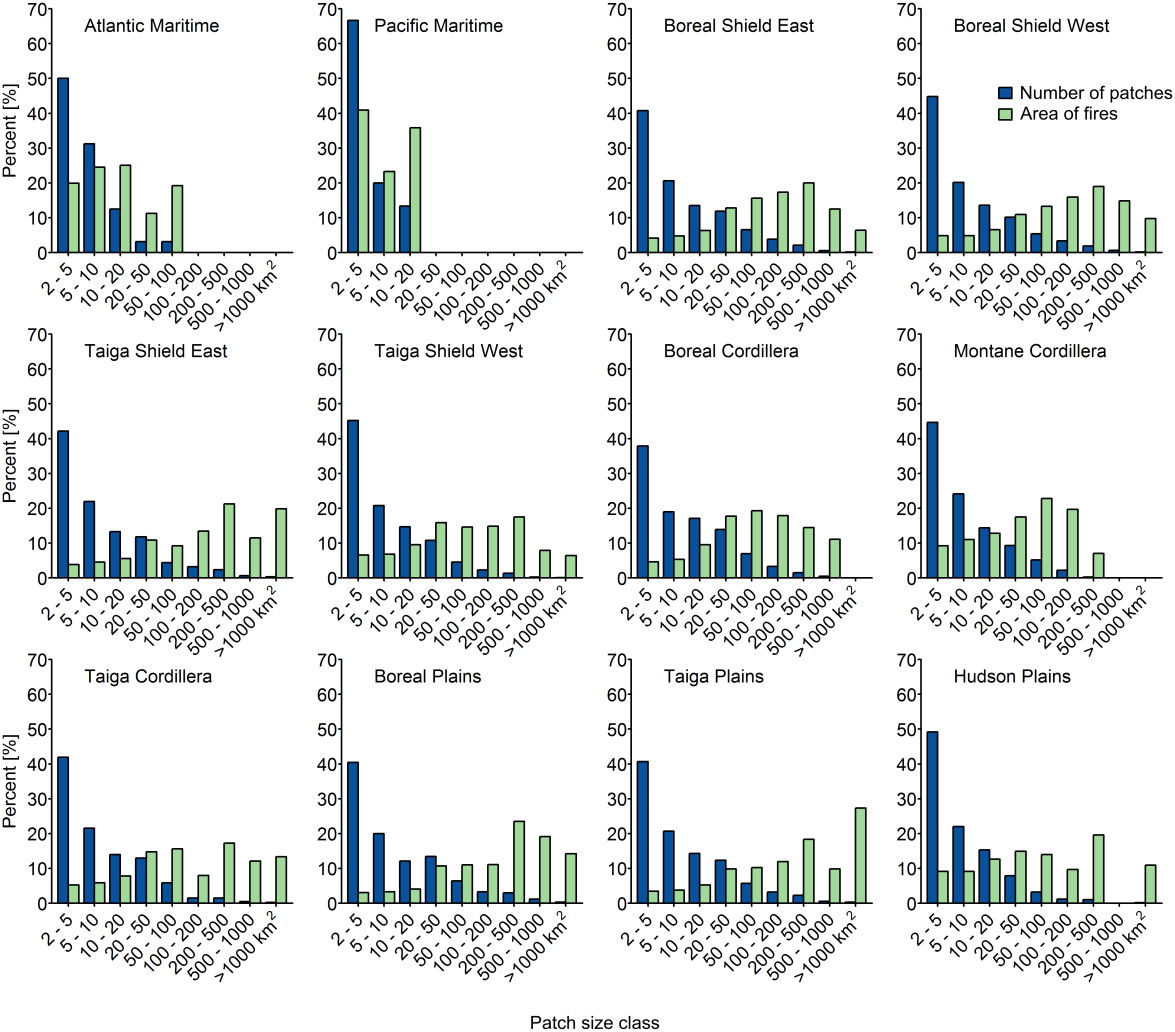
Burned area and number of burned patches, by patch size class.

Fig 7 shows the distribution of the number of burned patches and the total burned area over the entire analysis period and demonstrates the capacity of the Landsat time series approach to detect small fire patches. In all ecozones, small fire patches (2–5 km^2^) were most numerous; however, these small patches comprised only a small proportion of the total burned area in most ecozones. In contrast, only 3% of burned patches nationally were greater than 200 ha in size; however, these burned patches accounted for 63% of the total burned area. The Atlantic and Pacific Maritime ecozones showed evidence of fire regimes dominated by small burned patches. In contrast, the Boreal Shield East and West ecozones exhibit much larger variability in patch size than the other ecozones, with the greatest proportion of burned area occurring in patches that were 200–500 km^2^ in size. The distribution of burned area in the Taiga Plains is skewed towards larger burned patches, with the largest proportion of burned area (~30%) occurring in burned patches that were greater than 1000 km^2^ in size.

**Fig 8:**
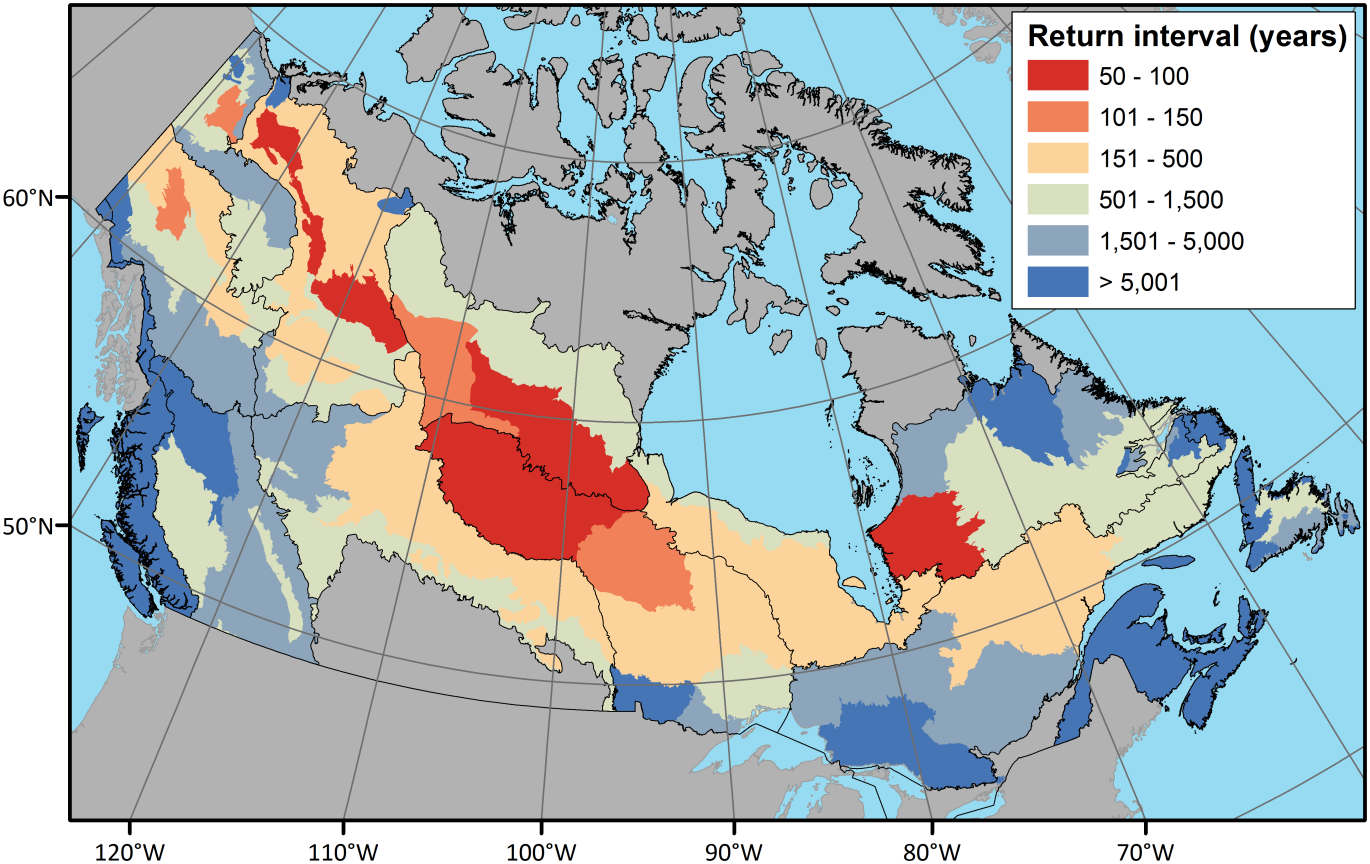
Estimated fire return interval within each ecoregion. Map generated in ArcGIS 10.1 (http://www.esri.com/software/arcgis/arcgis-for-desktop). Ecozone boundaries available via creative commons.

**Fig 9:**
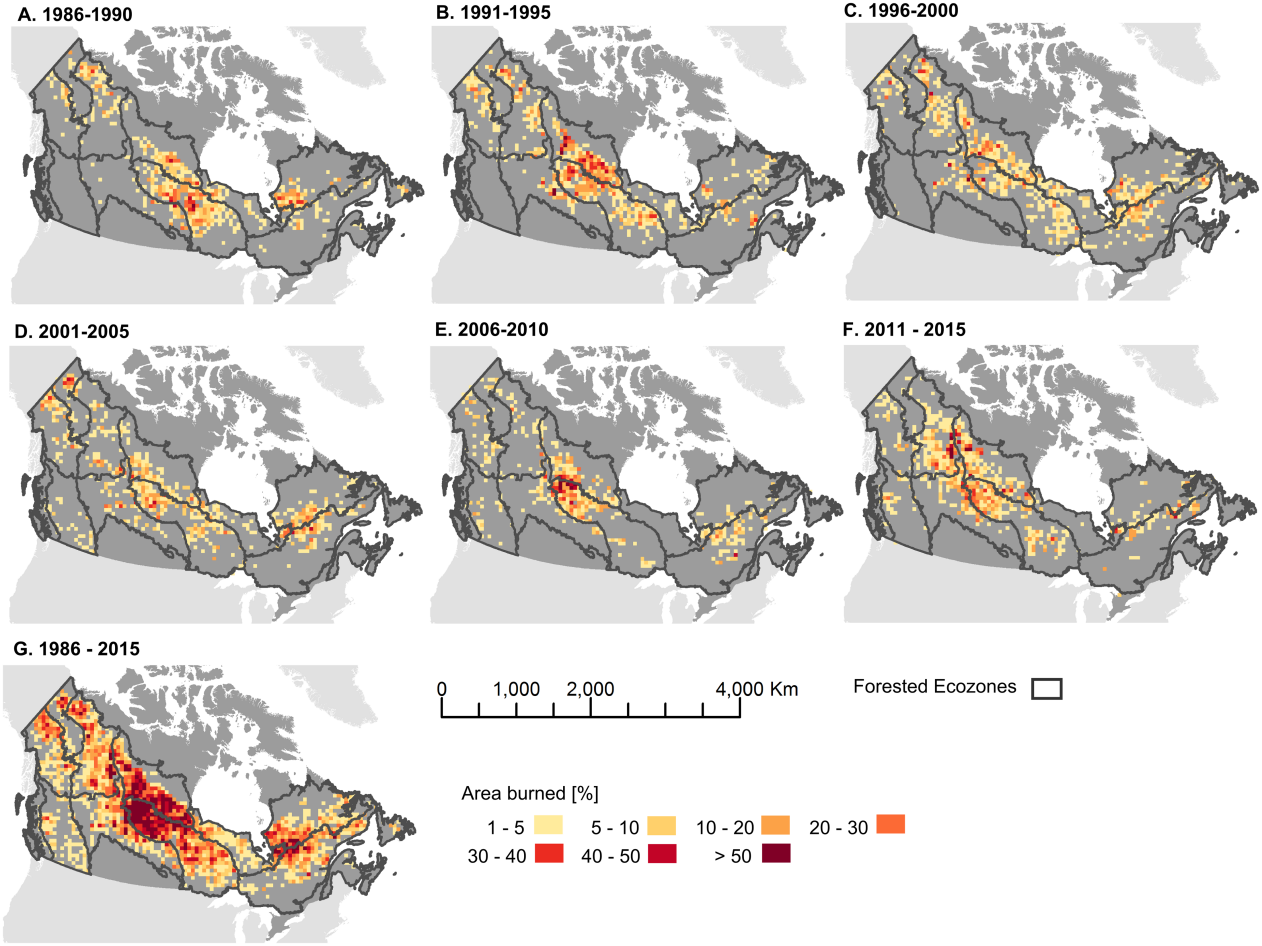
Percent area burned per 50 km × 50 km cell over the forested ecozones of Canada. Map generated in ArcGIS 10.1 (http://www.esri.com/software/arcgis/arcgis-for-desktop). Ecozone boundaries available via creative commons.

For each ecozone, we computed the mean fire return interval using exponential fitting (See Methods, Table 1). The Atlantic and Pacific Maritime ecozones have the longest mean fire return interval. In contrast, the Taiga Plains (188 years), Boreal Shield West (141 years), and Taiga Shield West (139 years), have the shortest mean fire return intervals. We also applied the exponential fitting methodology to the Canadian ecoregions, in order to provide a more detailed characterization of the fire return interval and highlight variability in return intervals within ecozones (Fig 8). For example, within the Boreal Shield West it is apparent that moving northwards the fire return interval decreases indicating fires are more common. The reverse is true for the Taiga Shield West which has a lower fire return interval at its northern range. Fire regimes were analysed over a larger spatial support region to enable analysis of spatial patterns. Analysis of the 50 × 50 km cells across the forested regions by 5-year epochs indicates the spatial and temporal variability of fire at the national scale, with the highest burn rates occurring in the 1991–1995 epoch (Fig 9).

## Discussion

Through a systematic and automated approach, we have analysed the spatio-temporal variability in burned area across Canada’s forested ecosystems for the past 30-years. Differences in fire activity between ecozones is a function of many competing factors, including fuel availability, fuel flammability, the probability of lightning strikes, climate, drought, and human influence [5,30]. Climatic factors, which vary regionally across Canada, are the primary factors controlling fuel availability, flammability, and lightning strikes [30,49]. The central and western boreal of Canada is drier and more prone to lightning strikes than the eastern boreal [5,30], resulting in burn rates that are more than three times higher in the Boreal Shield West than the Boreal Shield East, and more than two times higher in the Taiga Shield West than the Taiga Shield East between 1985–2015. The wet temperate climates and active fire suppression policies associated with proximity to human settlement in both the Pacific and Atlantic Maritime ecozones result in the extremely low burn rates observed in these ecozones, and high proportion of small patch sizes [14]. The Montane Cordillera also has a relatively low burn rate due to the combination of a wet climate, active fire suppression, and rough terrain that act as natural fire breaks [5]. Trends in the amount of burned area and their temporal trends were similar between the Boulanger et al. [9], homogeneous fire regime units and the ecozones across Canada, despite the regimes being derived from fire occurrence data, as opposed to the more generalised biotic and abiotic characteristics captured by the ecozones. Area burned varied markedly by fire regime unit with 3 of 16 showing significant trends. Similar locations of trends in western Canada for both ecozones and fire regime regionalization also suggest they both capture the same fire pattern behaviour with respect to area burned.

In addition to longitudinal trends in fire activity, latitudinal trends also exist as a function of human influence and climate. Public safety and resource protection lead to an emphasis on fire suppression in the southern regions of Canada resulting in low area burned along the southern fringes of the forested ecozones, while unmanaged forests in northern Canada are left to burn naturally [5], explaining the increase in fire activity from south to north in both the Boreal Shield East and West. The reverse was observed for the Taiga Shield East and West, however, with fire activity highest along the southern edge of the ecozones and lowest in the north. This may be due to limited fuel availability in the northern extents of these ecozones, where growing seasons are too short and cold to support notable tree growth.

The area burned across Canada can vary markedly from year to year due to variations in fire weather conditions (temperature, precipitation and drought). Above average burn years typically occur when conditions are hotter and drier than normal [50]. In 1989, 1995, and 2004, positive anomalies in area burned were observed across multiple ecozones, suggesting that broad climatic patterns contributed to these large fire years [51]. In addition to suitable climatic conditions, the annual area burned can be largely influenced by the presence or absence of several extremely large fires in unmanaged northern regions, such as three large fires in the Taiga Plains in 1989, whose area accounted for > 40% of the total burned area that year across Canada. Analysis of trends in area burned nationally also showed no relationship taking into account years with marked increases or decreases in the El Niño–Southern Oscillation index. Given century long fire return intervals in many of these ecozones, and large variability in the annual area burned, short-term trends over the past decade need to be taken with caution.

While large changes in fire weather are expected across the Canadian boreal [52,53], we observed few significant trends in burned area were over the entire period (1985–2015). This is consistent with the findings of other studies; Jolly et al. [3] found no significant change in fire weather in North American boreal regions from 1979 to 2013. Additionally, anomalous fire years during the Landsat record, such as 1989 and 1995, would prevent gradual changes in fire activity from being observed over the 30-year study period. As the trend analysis shows however, the last decade of the examined period (2006–2015) shows increasing trends in burned area and further analysis of additional years is critical to examine if these detected trends are indicative of a longer term trend. Increases in burned area in recent years is not unexpected given flammability is expected to increase across large areas of the Canadian boreal as climate changes, with proposed increases in burned area of 74–118% by the end of the century across Canada under a 3 × CO^2^ scenario [52]. Expected continuity of Landsat data will allow these changes in burned area to be accurately measured into the future [54].

Varying fire return intervals across Canada influence the age and structure of forest stands. Kneeshaw and Gauthier [55] demonstrated that western boreal stands have a lower proportion of old growth forests and stands > 200 years old than eastern boreal stands, due to the shorter fire return interval in the continental west. Where fire return intervals are very short, the initial cohort of trees can dominate stands until the next fire, while longer return intervals allow for the breakup of initial cohort and the formation of gaps, leading to more structurally complex forest stands [30]). In the Pacific Maritime, Daniels and Gray [14] found that long fire return intervals led to forest stands that were governed primarily by gap dynamics, not fire. In ecozones such as the Pacific and Atlantic Maritime, where fire is rare, estimating the return interval using 30 years of data requires cautious interpretation, as the presence or absence of rare fire events during this time period can significantly influence the estimated intervals. Daniels and Gray [14] suggested return intervals of 350 to thousands of years across the Pacific Maritime. Therefore, caution should be taken when interpreting the fire return intervals in ecozones with rare events.

Landsat-based assessments of burned area across Canada can augment historical fire information, such as CNFDB, which is a multi-source compilation of fire polygon data collected by fire management agencies across Canada. While compiling the CNFDB required years of data collection and coordination across management agencies, Landsat time-series analysis allows researchers to reconstruct disturbance history over the 30 m Landsat record for the entire landmass of Canada. Kasischke and Turetsky [56] noted a doubling in burned area between the 60s/70s and the 80/90s across boreal North America using the CNFDB, but acknowledged that this increase may have been caused by incomplete records in the CNFDB for the 60s/70s [5,57]. Consistent satellite-based estimates therefore can form baselines from the mid-1980’s to the present upon which to compare future areas burned across Canada. An additional advantage of the Landsat time series approach over the CNFDB is the spatially explicit estimates of area burned. Frequently, the CNFDB consists of generalized polygons that contain both burned and unburned forest patches as well as wetlands and waterbodies. In applications that aim to assess forest recovery following disturbance [58], it is difficult to isolate disturbed forest patches using the CNFDB alone. Therefore, Landsat-derived estimates of burned area enable analyses to focus on disturbed patches only, allowing a clearer assessment of forest recovery [59].

The CNFDB is an extremely valuable and irreplaceable data source for fire monitoring in Canada. The Landsat time series approach presented herein allows the precise area of burns to be determined; however, local knowledge and proactive monitoring by fire management agencies can provide information on ignition source, start date, and suppression efforts. This specific information is valuable for assessing fire cause and action taken and is missing from the Landsat time series approach applied herein. Further, while burned patches are derived in the Landsat time series approach, it is difficult to quantify the number of fires, as single fire patches can be detected as a series of many disconnected burned patches.

Practices implemented by provincial and territorial resource management agencies result in burned patches being grouped into fire events representing the life cycle of a given fire. These events often include unburned islands and water bodies, which increases the burned area associated with each fire event. Capture and tracking of fire events enables a detailed assessment of changes in the number of fires and fire sizes across Canada in a manner that is particularly informative for fire management and suppression efficacy point of view. Therefore, while the Landsat time series approach allows for a more detailed assessment of burned area, the CNFDB provides additional attributes for fire events, such as fire start and end date, causal agent, and suppression efforts. Besides differences in data sources, dissimilarities in methodological approaches explain the divergence in reported results (via consideration of patches) and those of previous studies (where events are considered). For example, Stocks et al. [5] found that fire events greater than 200 ha in size accounted for 97% of total burned area in Canada. In our study, with an emphasis on mapping of burned patches, we found that burned patches greater than 200 ha in size accounted for only 63% of the total area burned. This difference is likely the result of our analysis of patches rather than events. Looking forward, we thus foresee opportunities for the combination of temporal, geometric, and spectral information to aid in the informed spatial agglomeration of burn patches into fire events. Remotely sensed estimates of burned area provide an assessment of fire regimes that is based on the actual area burned and variability in this area over space and time, without interpretations related to fire management or suppression considerations. Consequently, considering both sources of fire information would be necessary to accurately map burned areas while also integrating information on fire seasonality, fire season length, causal agent, and suppression efforts (e.g., human versus lightning). When integrated, these data can provide comprehensive measures of the fire regime variability that can be modeled in a spatially explicit manner (e.g. [60]). The increasing capacity to capture within season satellite imagery at a high frequency will offer unique opportunities for coupling patch and event perspectives on fire mapping and the interpretation of regimes. Further, studies such as ours highlight the trade-offs in improved capture of spatial extent and uniformity of burned patches from higher resolution imagery versus depictions of a lower level of spatial detail (e.g., missing unburned island, inclusion of waterbodies within fire events).

The results of this study provide a spatially and temporally consistent representation of burned area from a single remote sensing data source, across Canada’s forested ecosystems, and a regionalisation of fire regimes, that can be used to inform both national monitoring programs and scientific applications. With recent significant temporal trends in burned area found during our 30-year study period, these results provide a critical baseline upon which future area burned can be compared. In addition, fusion of these data with more conventional fire event databases will allow a comprehensive examination of fire regimes across Canada. Given to the systematic nature of satellite based analysis, additional years of data can be incorporated as these data become available, allowing trends to be observed over a longer time period, in a near-real time enabled by data availability and computing. Additionally, with the launch of Sentinel 2 in 2014, which has similar imaging characteristics to the Landsat series [61], enhanced opportunities will exist for capturing within-year timing of burn events and provide improved opportunities for image gap filling via compositing processes [54].
